# Effects of Early Gait Training on Inpatient Frailty After Transcatheter Aortic Valve Implantation

**DOI:** 10.7759/cureus.63086

**Published:** 2024-06-25

**Authors:** Kenji Tsujimoto, Shinichi Watanabe, Tomokazu Kondo, Shohei Kawabata, Hiroyuki Okura

**Affiliations:** 1 Department of Cardiology, Gifu University Graduate School of Medicine, Gifu, JPN; 2 Department of Rehabilitation Medicine, Ichinomiyanishi Hospital, Aichi, JPN; 3 Department of Rehabilitation Medicine, Gifu University of Health Science, Gifu, JPN

**Keywords:** grip strength, walking velocity, gait training, frailty, transcatheter aortic valve implantation

## Abstract

Introduction: This study aimed to clarify the relationship between the number of days of early gait training and frailty in in-hospital patients undergoing transcatheter aortic valve implantation (TAVI) for aortic stenosis, focusing on the Clinical Frailty Scale (CFS) and clinical laboratory data.

Methods and Results: Sixty-nine patients admitted to the Ichinomiya West Hospital from November 1, 2019 to November 30, 2023 were included in the study. Of the 69 patients, those who started gait training on postoperative day 0 or 1 were defined as the early gait training group and those who started gait training later than postoperative day 1 were defined as the delayed gait training group. There was a significant difference in the number of days to gait training initiation, which was 3.9 days in the delayed gait training group and 0.9 days in the early gait training group. The early gait training group started early mobilization and had a significantly shorter postoperative hospital stay than the delayed gait training group. Clinical laboratory data showed that walking speed was significantly faster and grip strength was significantly higher in the early group. The number of days to gait training initiation was an independent predictor of changes in CFS scores.

Conclusion: Early gait training in patients after TAVI may predict early improvements in physical function and movement, shorter hospital stay, and frailty at discharge.

## Introduction

In recent years, frailty has been reported to be a major cause of mortality and rehospitalization in patients with heart disease [1]. Patients with frailty have a poor prognosis after transcatheter aortic valve implantation (TAVI), a treatment for aortic stenosis [2]. Moreover, postoperatively, 6.6% of patients develop heart failure within 1 year or are classified as New York Heart Association class ≥3 at 1 year, with frailty as an independent predictor of the onset of heart failure [3].

The prevalence of frailty was reported to be up to 68% in a prospective cohort study of patients treated with TAVI or surgery [4]. Furthermore, in Japan, TAVI is performed in high-risk or inoperable patients as an alternative to surgical treatment. By contrast, TAVI is minimally invasive and allows for early functional and movement training. Gait training can be expected to prevent or reduce frailty through early rehabilitation. However, few studies have reported the effects of early gait training on in-hospital patient frailty after TAVI. Gait training is possible as early as 4 hours after TAVI if no intra- or postoperative complications are observed [5]. Therefore, this study aimed to analyze the effect of early gait training on frailty during hospitalization after TAVI.

## Materials and methods

**Figure 1:**
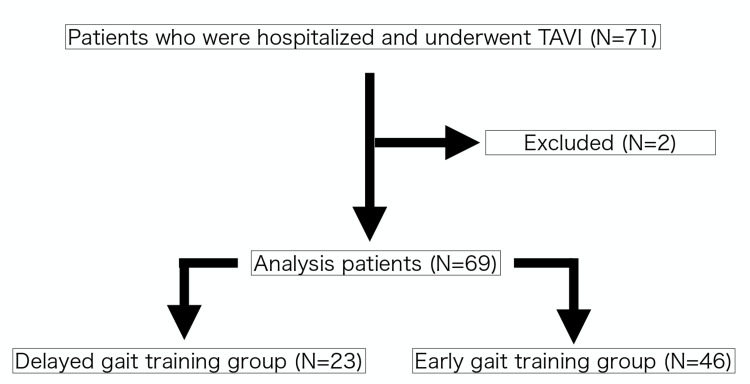
Flowchart of analysis participants

Seventy-one patients who were hospitalized at Ichinomiya-Nishi Hospital and underwent TAVI for aortic stenosis between November 1, 2019, and November 30, 2023, were included in the study. Two patients who had difficulty walking were excluded from the analysis, resulting in 69 participants. Patients who started gait training on postoperative day 0 or 1 were defined as the early gait training group and those who started gait training later than postoperative day 1 were defined as the delayed gait training group (Figure 1).

Rehabilitation during hospitalization was performed according to the protocol at Ichinomiya West Hospital, with 50-m gait training on postoperative day 0 or 1 after TAVI and 200-m gait training on postoperative day 2. The criteria for starting stepping up gait training were as follows: no chest pain, severe shortness of breath, severe fatigue with a Borg score > 13, dizziness, or lightheadedness, cyanosis, pallor, or coldness, no exercise-induced increase in arrhythmias or rhythmic changes leading to atrial fibrillation, no exercise-induced ischemic electrocardiogram, no excessive changes in blood pressure caused by exercise, no increase in heart rate by >30 bpm due to exercise, and no decrease in oxygen saturation below 90% due to exercise [6]. In addition to the preoperative and pre-discharge Clinical Frailty Scale (CFS) assessments, clinical laboratory data were recorded at the same institution.

Patient demographic and clinical data, such as age, sex, outcomes, long-term care insurance, reason for hospitalization, number of pre- and postoperative days, surgery type, and drug information, were extracted from medical records. The number of rehabilitation units per day was calculated by adding the number of rehabilitation units provided by the physical and occupational therapists. The number of days of early mobilization, days of gait training, and CFS scores were extracted from the rehabilitation medical records. In the delayed gait training group, the causes of delayed gait training were identified based on medical information. The frailty index is a typical measure for evaluating frailty. However, it comprises a large number of items and is complicated to calculate, making it difficult to use in clinical situations. However, the CFS score is strongly correlated (r=0.80) with the frailty index; furthermore, the risk of mortality and institutionalization increases with worsening CFS scores [7]. Using the CFS, frailty is rated with a score of 1 to 9 based on the severity of symptoms and activities of daily living (ADL), with scores of 1 to 3 and 5 to 9 being indicative of “not frail” and “frail,” respectively. In this study, a CFS score ≥ 5 was considered indicative of frailty [7], and the presence of preoperative frailty and changes in CFS scores before surgery and discharge from the hospital were evaluated.

Furthermore, the Mini-Mental State Examination was conducted by a physical therapist and an occupational therapist to evaluate cognitive function, and 5-meter walk, 10-meter walk speed, short physical performance battery, grip strength, upper arm circumference, and lower leg circumference tests were conducted to evaluate physical function. In the 5-meter walk test, the 5-meter walking distance was measured at a comfortable walking speed, and the walking time was assessed once. In the 10-meter walking speed test, the acceleration and deceleration intervals were 3 meters before and after the 16-meter walking distance, and the measurement interval was 10 meters. The maximum walking speed was measured twice, and the fastest walking speed was adopted. Grip strength was measured twice in a standing posture on each side, and the maximum value of all measurements was used.

The Controlling Nutritional Status score and albumin levels were measured in blood samples collected for nutritional evaluation. C-reactive protein level was measured to assess inflammation, hemoglobin to assess anemia, brain natriuretic peptide to assess the severity of heart failure, and blood urea nitrogen, creatinine, and glomerular filtration rate to assess renal function.

Based on echocardiographic results, we measured the left ventricular ejection fraction to evaluate left ventricular contractility, and we measured early diastolic mitral annular velocity and early diastolic mitral annular velocity/systolic mitral annular velocity to evaluate left ventricular diastolic performance. To evaluate the severity of aortic stenosis, we extracted data on the maximum aortic velocity, aortic mean pressure gradient, and aortic valve area.

In the statistical analysis, characteristics were compared between the delayed and early gait training groups using either the unpaired t-test or χ2 test. Clinical laboratory data were compared between the two groups using the unpaired t-test. In addition, univariate logistic regression analysis was conducted to examine the effect of early mobilization and early gait training on changes in CFS scores, using the two groups of patients with worsened and non-worsened CFS scores as objective variables and the number of days of early mobilization and early gait training as explanatory variables. Furthermore, multivariate logistic regression analysis was conducted to exclude the effects of age and sex, both of which were adjustment factors. All analyses were performed using JMP (version 13.0; SAS Institute, Cary, NC, USA), and a P-value < 0.05 was considered statistically significant. This study was conducted in accordance with the Declaration of Helsinki and the Ethical Guidelines for Medical Research Involving Human Subjects; moreover, it was approved by the Ethical Review Committee of Ichinomiya West Hospital (approval number: 2023057).

## Results

There were no significant differences in mean age or sex. The mean number of postoperative days was 8.6 days in the early gait training group, which was a significantly shorter duration than that in the delayed gait training group. The early gait training group performed mobilization significantly earlier than the delayed gait training group. In the delayed and early gait training groups, gait training was initiated 3.9 and 0.9 days postoperatively, respectively (p=<0.001). Of the patients in the delayed gait training group, nine developed new arrhythmia intra- or postoperatively, three developed hemodynamic instability during rehabilitation training and required drug adjustment, one was placed on a ventilator, and 13 required bed rest under medical supervision. In addition, seven patients showed reluctance and could not cooperate with gait training, and gait training was delayed to a further date in two patients owing to restless behavior. Preoperative CFS assessment showed that the prevalence of frailty was 9 patients (39%) in the delayed gait training group and 15 patients (33%) in the early gait training group, with no significant differences (Table 1).

**Table 1:** Comparison of characteristics among all patients a: Unpaired t-test, b: χ2 test p-value <0.05 was considered statistically significant TF: transfemoral, TA: transapical, CFS: Clinical Frailty Scale, β blocker: beta blocker, ARB: Angiotensin Ⅱ Receptor Blocker, ACE: Angiotensin Converting Enzyme inhibitor, ARNI: Angiotensin Receptor Neprilysin Inhibitor, MRA: Mineralocorticoid Receptor Antagonist, Ca-blocker: Calcium Channel Blocker, SGLT2 inhibitor: Sodium-Glucose Transporter 2 Inhibitor

	Delayed gait group (N=23)	Early gait group (N=46)	p-value
Age(years) (Mean ± SD) ^a^	86.2 ± 3.6	84.5 ± 4.8	0.120
Gender(male/female) (N) ^b^	7 / 16	16 / 30	0.791
Outcome(death/rehospitalization/discharge) (N) ^b^	3 / 4 / 16	3 / 5 / 38	0.453
Long-term care insurance(yes/no) (N) ^b^	11 / 12	17 / 29	0.442
Reason for hospitalization(expected/exacerbation) (N) ^b^	17 / 6	37 / 9	0.550
Preoperative days(days) (Mean ± SD) ^a^	10.0 ± 10.7	8.8 ± 9.3	0.406
Postoperative days(days) (Mean ± SD) ^a^	13.8 ± 6.6	8.6 ± 2.8	<0.001
Preoperative rehabilitation units/day(days) (Mean ± SD) ^a^	1.8 ± 0.8	2.0 ± 1.2	0.281
Postoperative rehabilitation units/day(days) (Mean ± SD) ^a^	2.8 ± 0.8	2.6 ± 1.1	0.426
Days to start early mobilization(days) (Mean ± SD) ^a^	1.8 ± 1.2	0.9 ± 0.3	<0.001
Days to start gait training(days) (Mean ± SD) ^a^	3.9 ± 5.3	0.9 ± 0.2	<0.001
Surgery type(TF/TA) (N) ^b^	21 / 2	42 / 4	1.000
Preoperative frailty (N, %) ^b^	9 (39%)	15 (33%)	0.603
Beta-blocker (N, %) ^b^	8 (35%)	20 (43%)	0.605
ARB/ACE/ARNI (N, %) ^b^	12 (52%)	24 (52%)	1.000
Loop diuretics (N, %) ^b^	9 (39%)	21 (46%)	0.797
MRAs (N, %) ^b^	7 (30%)	13 (28%)	1.000
Inotropic (N, %) ^b^	1 (4%)	1 (2%)	1.000
Tolvaptan (N, %) ^b^	3 (13%)	8 (17%)	0.740
Ca channel blockers (N, %) ^b^	12 (52%)	20 (43%)	0.610
Nicorandil (N, %) ^b^	0 (0%)	2 (4%)	0.549
Nitrates (N, %) ^b^	1 (4%)	1 (2%)	1.000
Amiodarone (N, %) ^b^	5 (22%)	5 (11%)	0.283
SGLT2 inhibitors (N, %) ^b^	2 (8%)	10 (22%)	0.312

In the 5-meter walk test, the walking time was significantly shorter in the early gait training group (6.3 s) (p=0.046), and in the 10-meter walk speed test, walking speed was significantly faster in the early gait training group (0.9 m/s) than in the late training group (p=0.045). The grip strength was significantly lower in the delayed gait training group (16.0 kg) than in the early gait training group (18.8 kg) (p=0.030) (Table 2).

**Table 2:** Post-TAVI clinical laboratory data p-value <0.05 was considered statistically significant MMSE: Mini-Mental State Examination, SPPB: Short physical performance battery, CONUT: controlling nutritional status, Alb: albumin, BUN: blood urea nitrogen, CRE: creatinine, CRP: C-reactive protein, Hb: hemoglobin, BNP: brain natriuretic peptide, GFR: glomerular filtration rate, EF: ejection fraction, E/e': early filling, velocity/E wave with relaxation velocity on tissue doppler, E/A: early filling velocity / atrial filling velocity, AV peak V: aortic valve area velocity, AV mean PG: aortic valve mean pressure gradient, AVA: aortic valve area

	Delayed gait group (N=23)	Early gait group (N=46)	
	Mean±SD		p-value
MMSE (score)	24.9 ± 3.8	25.7 ± 4.2	0.375
5m walk test(s)	7.9 ± 3.2	6.3 ± 2.1	0.046
10m walk speed(m/s)	0.8 ± 0.3	0.9 ± 0.3	0.045
SPPB	7.6 ± 2.5	9.2 ± 2.7	0.114
Grip strength (kg)	16.0 ± 7.5	18.8 ± 7.9	0.030
Upper arm circumference (cm)	24.5 ± 4.4	23.6 ± 4.1	0.347
Lower Leg Circumference (cm)	30.4 ± 4.1	29.5 ± 3.5	0.185
CONUT (score)	4.3 ± 2.5	3.9 ± 2.1	0.589
Alb (g/dl)	3.2 ± 0.6	3.4 ± 0.4	0.132
BUN (mg/dl)	23.2 ± 7.9	21.0 ± 8.5	0.135
CRE (mg/dl)	1.0 ± 0.4	0.9 ± 0.3	0.550
CRP (mg/dl)	1.6 ± 1.8	2.3 ± 5.0	0.990
Hb (g/dl)	10.9 ± 1.7	11.1 ± 1.6	0.302
BNP (pg/ml)	195.3 ± 307.1	118.6 ± 136.7	0.165
GFR (ml/min/1.73m^2^)	50.1 ± 19.0	54.5 ± 17.9	0.288
EF (%)	63.1 ± 14.7	63.1 ± 9.8	0.503
E/e'	27.5 ± 10.6	24.1 ± 10.2	0.161
E/A	1.0 ± 0.8	0.7 ± 0.2	0.181
AV peak V (m/s)	2.8 ± 3.1	2.4 ± 0.6	0.624
AV mean PG (mmHg)	10.8 ± 6.6	12.9 ± 7.2	0.276
AVA (cm^2^)	1.5 ± 0.5	1.7 ± 1.9	0.631

Univariate logistic regression analysis with the number of days of gait training as the explanatory variable showed that the number of days to gait training initiation (odds ratio [OR]=1.564, p=0.012) was a significant predictor. Furthermore, multivariate logistic regression analysis, with age and sex as adjustment factors, showed that the number of days to gait training initiation (OR=2.239, p=0.043) was an independent predictor (Table 3).

**Table 3:** Effects of days to early mobilization and gait training initiation on changes in CFS scores a: univariate logistic regression analysis, b: multivariate logistic regression analysis p-value <0.05 was considered statistically significant

	Odds ratio	95%Cl	p-value
Days to early mobilization ^a^	1.668	0.867-3.207	0.125
Days to early mobilization (adjusted by age and sex) ^b^	2.456	0.691-8.730	0.165
Days to gait training initiation ^a^	1.564	1.121-2.598	0.012
Days to gait training initiation (adjusted by age and sex) ^b^	2.239	1.027-4.878	0.043

## Discussion

In this study, we examined the effect of early gait training initiation on frailty during hospitalization by dividing patients into two groups: the delayed and early gait training groups. Early postoperative gait training can predict the prevention or reduction of frailty, which is a state of reversible decline in physical and mental function. The patients in our study did not develop any new complications during or after surgery and were discharged from the hospital without any sudden changes or worsening of their condition during hospitalization. Furthermore, the patients in the early gait training group began mobilization significantly earlier than those in the delayed gait training group, and in terms of physical function, their walking speed was faster, and their grip strength was significantly higher at the time of discharge, enabling a reduction in the number of postoperative hospitalization days. Walking speed and grip strength are included in the frailty index and Fried’s diagnostic criteria for frailty [8]. Walking speed has been reported to be a highly sensitive marker for assessing frailty [9].

However, 13 of the 69 participants in this study required a period of bed rest for medical management owing to intra- and postoperative effects, and gait training initiation was delayed. The start of early mobilization was also delayed for the same reasons. Therefore, the start of early mobilization was also significantly delayed in the delayed gait training group compared with the early gait training group. The reason for the delay in walking was that seven patients did not cooperate in gait training, and two patients experienced restlessness. Thus, we believe that, in the future, in addition to physical function and movement training, a sufficiently detailed explanation of postoperative rehabilitation, adjustment of the environment around the bed, and consideration of hospitality are necessary during preoperative rehabilitation.

These delays, which increased the number of days to early mobilization and gait training initiation, resulted in a decrease in grip strength, a decrease in walking speed, and a worsening of CFS scores. Morimoto et al. reported that a delay in early mobilization increases the prevalence of sarcopenia and that a delay in gait training initiation correlated with a decrease in grip strength and walking ability [10]. It has also been speculated that muscle weakness and reduced walking ability affect the level of rest and activity in the hospital, which may contribute to further disability. Nevertheless, early mobilization and gait training reportedly contribute to improved ADL and walking ability [11]. We hypothesized that the CFS score would be maintained or improved in the early gait training group, whereas a decline in physical function and worsening of CFS scores would be observed in the delayed gait training group.

We believe that these physical functions were among the factors that influenced the reduction in the number of postoperative hospitalization days in the early gait training group. Walking is the most basic ADL at home. Furthermore, a survey of elderly patients with heart disease revealed that among ADLs, mobility had a significant impact on the discharge destination being the patients’ home [10,12]. The average length of hospital stay for post-TAVI patients is 11 days [13]. Therefore, we believe that starting gait training on day 0 or 1 was effective in our study participants and enabled their early discharge from the hospital.

Limitations

This study was conducted at our institution based on the clinical records. The study included elderly patients with cardiac disease, and it is possible that confounding factors that were not analyzed influenced the results. In addition, the number of postoperative hospitalization days may not solely depend on the patient’s physical function, and other factors were not considered in this study. Moreover, the clinical laboratory data in this study were obtained only from the results of pre-discharge evaluations, and it is necessary to confirm the validity of this study by increasing the number of cases and conducting a longitudinal study.

## Conclusions

The present study enabled us to confirm the effect of delaying the start of gait training and the efficacy of early initiation of gait training after TAVI. Early gait training was effective in preventing frailty exacerbation in patients with changes in CFS scores. These findings may contribute to the development of a post-TAVI training program for the early improvement of physical function and movement, shortening of hospital stays, and frailty prediction at discharge.
